# Efficacy of Oral Tranexamic Acid Versus Combined Oral Contraceptives for Heavy Menstrual Bleeding

**DOI:** 10.7759/cureus.19122

**Published:** 2021-10-29

**Authors:** Sundus Rahman, Fatima S Khan, Kashif A Samin, Nighat Afridi, Moiz Ahmed

**Affiliations:** 1 Department of Obstetrics and Gynecology, Combined Military Hospital (CMH), Peshawar, PAK; 2 Department of Obstetrics and Gynecology, Pak Emirates Military Hospital (PEMH), Rawalpindi, PAK; 3 Department of Family Medicine, Khyber Medical University, Peshawar, PAK; 4 Department of Medicine, Jinnah Postgraduate Medical Centre, Karachi, PAK

**Keywords:** coc pills, levonorgestrel, contraceptives, menorrhagia, heavy menstrual bleeding, tranexamic acid, folic acid, placebo, diabetes mellitus

## Abstract

Introduction

Heavy menstrual bleeding (HMB) is characterized by high blood loss (>80 mL per cycle) at regular menstrual intervals. It can have an impact on a woman's bodily, mental, and/or material well-being. The etiology is varied and can be local, systemic, or iatrogenic. The occurrence of HMB is between 4% and 27%, depending on objective menstrual bleeding measurements and on high estimates based on subjective bleeding measures. This study was conducted to assess the efficacy of oral tranexamic acid versus combined oral contraceptive (COC) pills in the management of excessive menstrual bleeding.

Methodology

A comparative study was conducted at the Obstetrics and Gynecology Department of Combined Military Hospital Peshawar, Pakistan, from October 2020 to March 2021. Women aged above 18 years who presented with heavy menstrual bleeding (HMB) were included in the study. The exclusion criteria included all women with contraindications to the use of tranexamic acid, such as lactating mothers, pregnancy, use of oral contraceptives or steroids, history of renal malfunction or stroke, family history of thromboembolic disease, and ovarian or endometrial carcinoma. Patients with diagnosed leiomyomas with a size between >1 and 10 cm were included in the study. Women were allocated randomly into group A who received oral tranexamic acid 3.9-4 g per day or group B who received oral COC pills containing a combination of ethinyl estradiol 30 μg and norgestrel 0.3 mg. The efficacy of treatment was considered successful if there was a mean reduction in menstrual blood loss that was significantly greater than the baseline values.

Results

There were 178 patients in total, with 89 patients in each group. It was found that both oral tranexamic acid and combined oral contraceptives were equally effective in reducing the mean blood loss among patients and there was no statistical difference observed between the two groups. Upon stratification, it was found that both treatment groups were highly effective in younger age groups. Similarly, there was no significant difference in efficacy with respect to diabetes mellitus or hypertension. However, in individuals with leiomyomas, efficacy was significantly higher in patients who were in group B (combined oral contraceptives) (p = 0.004), and 46.1% of women in group A and 60.6% of women in group B did not experience any discomfort.

Conclusion

The current study revealed that both oral tranexamic acid and COC pills were equally effective in reducing the mean blood loss among patients with HMB. It was further found that the efficacy of both therapies was significantly higher in younger age groups. The efficacy of therapy was significantly reduced with the increasing age of the patient. Moreover, it was found that patients with leiomyomas benefitted more significantly from COC pills. There were no severe adverse effects reported in the study. However, future researches can explore the long-term side effects of both therapies.

In short, both therapies were comparable in terms of efficacy and safety. Heavy menstrual bleeding can negatively impact a woman, emotionally and physically. Therefore, it is encouraged that physicians use their expert judgment while prescribing either oral tranexamic acid or COC pills to patients with HMB.

## Introduction

Heavy menstrual bleeding (HMB) is characterized as an excessive amount of bleeding (>80 mL per cycle) that occurs at either regular or irregular intervals [1]. It can have an impact on a woman's bodily, mental, emotional, and/or material well-being [2]. Its etiology is varied and can be local, systemic, or iatrogenic. No specific etiology was identified in half of all cases, and such cases are classified as idiopathic [3]. HMB diagnoses are often based on the affected individual's perspective and its effect on the everyday lives of those who experience it. Often, women consider treatment because of the adverse consequences of menorrhagia on their health-related living conditions (e.g., daily life limitations, responsibilities of their employment, and social interactions) [4-8]. The reduction of monthly bleeding can be effectively achieved by the usage of hormonal medicines levonorgestrel-releasing intrauterine system or combined oral contraceptives (COCs), nonsteroidal anti-inflammatory medications, and surgery. However, the application of these treatments can be limited by relative contraindications, efficacy disparities, and harmful or unwanted impacts on fertility [9]. COC pills have been used substantially for the treatment of HMB; however, there is very limited evidence of their efficacy.

Antifibrinolytic tranexamic acid has been found to be beneficial for clinical use for HMB. It has been used in several European states for almost 40 years for this indication. In the USA, however, the treatment of menorrhagia was not authorized until 2009 for tranexamic acid [5]. HMB can lead to anemia, which can be difficult to handle by ongoing iron treatment, as well as social drawbacks. Leiomyomas and dysfunctional uterine bleeding have been the most frequent gynecological causes of HMB [10]. Menorrhagia can be caused by bleeding disorders such as von Willebrand disease and platelet damage in other women. However, more than a third of women who have HMB hysterectomy have their anatomically normal uterus taken out [11], while 80% of women treated for menorrhagia have no anatomical pathology.

In Pakistan, the majority of the women do not consider opting for medical treatments against HMB either due to inaccessibility to a nearby health clinic or lack of awareness. Moreover, there is a lack of studies comparing the efficacy of tranexamic acid with other alternative medications. Thus, the rationale behind the study was to add useful data to the existing literature by assessing the efficacy of tranexamic acid in comparison with combined oral contraceptive pills for the management of HMB. This study will help us establish whether COC pills are a better option for HMB as compared with oral tranexamic acid.

## Materials and methods

A comparative study was conducted at the Obstetrics and Gynecology Department of Combined Military Hospital Peshawar, Pakistan, from October 2020 to March 2021. Women aged above 18 years who presented with heavy menstrual bleeding (HMB) were included in the study. All women who presented with heavy bleeding for four or more consecutive days over a period of the last six months were diagnosed with HMB. The exclusion criteria included all women with contraindications to the use of tranexamic acid, such as lactating mothers, pregnancy, use of oral contraceptives or steroids, history of renal malfunction or stroke, family history of thromboembolic disease, and ovarian or endometrial carcinoma, were excluded from the study. Patients with diagnosed leiomyomas with a size between >1 and 10 cm were included in the study.

The study was approved by the ethical review board (reference number: F-2/IRB/5465/CMH). Women were allocated randomly into group A who received oral tranexamic acid 3.9-4 g per day or group B who received oral COC pills containing a combination of ethinyl estradiol 30 μg and norgestrel 0.3 mg. The women in group A were prescribed tranexamic acid three times per day starting from day one to day five of the menstrual cycle. Group B (COC) was prescribed three weeks of active pills and one week of placebo pills. If patients complained of severe gastrointestinal symptoms refractory to antiemetic medication, the test drug was stopped. However, in the present study, no severe adverse effects were reported.

All women who participated in the study were requested to ship their used sanitary napkins for the analysis. The labeled bags were provided to each participant, which then was sent for quantification of menstrual blood loss. The volume of blood was determined by an experienced technician who was blinded to the objectives of the study. The measurement of menstrual blood was based on the alkaline hematin method [12]. The efficacy of treatment was considered successful if there was a mean reduction in menstrual blood loss that was significantly greater than the baseline values. Then, the number of times the pads were used over the course of five days was utilized to determine the improvement in symptoms of HMB in the respective groups. Medication compliance was determined by the authors by reviewing patients' diaries and assessing the number of tablets. Non-adherent patients were excluded from the final analysis.

The Statistical Package for the Social Sciences (SPSS) version 22 was used to perform the data analysis. Qualitative variable efficacy, hypertension, and diabetes mellitus were measured as frequency and percentage. The mean and standard deviation of the quantitative variable age were calculated. The efficacy analysis was performed, comparing the menstrual blood levels at baseline and menstrual blood at one treatment cycle. The efficacy was compared in both groups. Stratification was used to control effect modifiers, including age, high blood pressure, diabetes mellitus, and leiomyomas, in both groups. The layered chi-squared test was applied. The significance level was set at p ≤ 0.05.

## Results

There were 178 patients in total, with 89 patients in each group. The patients in group A (oral tranexamic acid) had a mean age of 32.6 ± 4.1 years, whereas the patients in group B (combined oral contraceptives) had a mean age of 28.6 ± 3.2 years. In our study, 57 (32.02%) patients had hypertension, whereas 60 (33.71%) patients had diabetes mellitus. About 50 (28.1%) women had leiomyomas. The distribution of baseline parameters according to the treatment group is demonstrated in Table 1.

**Table 1:** Baseline Parameters of Group A (Tranexamic Acid) and Group B (Combined Oral Contraceptives)

Baseline Parameters	Group A (Tranexamic Acid)	Group B (Combined Oral Contraceptives)	P-Value
Mean age (years)	32.6 ± 4.1	28.6 ± 3.2	0.541
Age groups			
18–30 years	65 (73.03%)	63 (70.1%)	0.73
31–45 years	24 (26.9%)	26 (29.2%)	
Comorbidities			
Hypertension			
Yes	30 (33.7%)	27 (30.3%)	0.438
No	59 (66.3%)	52 (69.6%)	
Diabetes mellitus			
Yes	28 (31.5%)	32 (35.9%)	0.525
No	61 (68.5%)	57 (64.04%)	
Leiomyomas			
Present	22 (24.7%)	28 (31.4%)	0.317
Absent	67 (75.2%)	61 (65.5%)	

It was found that both oral tranexamic acid and combined oral contraceptives were equally effective in reducing the mean blood loss among patients and there was no statistical difference observed between the two groups (Table 2). 

**Table 2:** Efficacy of Group A (Tranexamic Acid) Versus Group B (Combined Oral Contraceptives)

Blood Loss (mL)	Group A (Tranexamic Acid)	Group B (Combined Oral Contraceptives)	P-Value
<80 mL	73 (82.02%)	62 (69.66%)	0.077
>80 mL	16 (17.98%)	27 (30.34%)	

Upon stratification, it was found that both treatment groups were highly effective in younger age groups (p = 0.361). Similarly, there was no significant difference in efficacy with respect to diabetes mellitus or hypertension. However, in individuals with leiomyomas, efficacy was significantly higher in patients who were in group B (combined oral contraceptives) (p = 0.004) (Table 3). 

**Table 3:** Stratification of Demographic and Clinical Parameters According to the Treatment Group and Efficacy (Blood Loss < 80 mL)

Variables	Group A (Tranexamic Acid, <80 mL)	Group B (Combined Oral Contraceptives, <80 mL)	P-Value
Age group			
20–30 years	58 (79.4%)	53 (85.48%)	0.361
30–45 years	15 (20.5%)	9 (14.50%)	
Hypertension			
Yes	20 (27.3%)	19 (30.7%)	0.678
No	53 (72.6%)	43 (69.3%)	
Diabetes mellitus			
Yes	13 (17.8%)	16 (25.8%)	0.259
No	60 (82.1%)	46 (74.1%)	
Leiomyomas			
Present	8 (10.9%)	19 (30.6%)	0.004
Absent	65 (89.04%)	43 (69.3%)	

The most common adverse effect reported was headache, followed by gastrointestinal symptoms including nausea, vomiting, and diarrhea. Individuals on combined oral contraceptive (COC) pills more frequently complained of headaches than those on tranexamic acid [30 (33.7%) versus 22 (24.7%)], and 46.1% of women in group A and 60.6% of women in group B did not experience any discomfort (Table 4).

**Table 4:** Distribution of Self-Reported Adverse Effects According to the Treatment Group

Adverse Effects	Group A (Tranexamic Acid)	Group B (Combined Oral Contraceptives)
Gastrointestinal symptoms	14 (15.7%)	18 (20.2%)
Headache	22 (24.7%)	30 (33.7%)
Musculoskeletal pain	12 (13.4%)	6 (6.7%)
No complication	41 (46.1%)	54 (60.6%)

## Discussion

Heavy menstrual bleeding (HMB) affects between 4% and 27% of women, with low estimated numbers centered on objective monthly bleeding measures and high estimates based on subjective bleeding readings [1]. Nearly half of the women who are tested for HMB do not have any underlying organic pathology [10]. Uterine fibroids and polyps are among the most commonly reported etiologies among those whose underlying cause can be determined [13].

HMB is a significant indication of morbidity. The authors of the current study presented a comparative study evaluating the efficacy of oral tranexamic acid versus combined oral contraceptives (COCs) among women with HMB. No similar study has been done in Pakistan; therefore, the study adds clinically important data to the existing literature. Since the efficacy of tranexamic acid in reducing blood loss during the menstrual period is well established, the authors explored an alternative treatment for HMB for patients belonging to a resource-restraint region, i.e., Pakistan. The study reported that both treatments successfully reduced blood loss during the menstrual cycle. Moreover, in patients with leiomyomas, COC was significantly more effective in reducing blood loss to <80 mL (p = 0.004) as compared with oral tranexamic acid. Moroni et al. published a systematic review in 2015 reviewing the efficacy of COC pills for the treatment of abnormal uterine bleeding in patients with leiomyomas. However, the review yielded no concrete evidence in favor of COC pills [14]. Subsequently, in the year 2020, Malik et al. explored the role of levonorgestrel intrauterine device and norethisterone for the treatment of menorrhagia. The authors revealed that both treatments were effective in the treatment of HMB; however, the levonorgestrel-releasing intrauterine device was significantly more effective and had higher patient satisfaction as compared with norethisterone [15].

In a study by Srivasths et al., it was found that both tranexamic acid and combined oral contraceptives significantly improved symptoms of menorrhagia (p < 0.05) [16]. Previously in 2004, the combined use of tranexamic acid and contraceptive pills was associated with acute myocardial infarction [17]. Milson and Rybo evaluated the efficacy of flurbiprofen, tranexamic acid, and levonorgestrel for the treatment of menorrhagia. They reported that menstrual blood loss was lowered by all three forms of therapy. However, the efficacy of levonorgestrel-releasing intrauterine contraceptive device was significantly greater than both tranexamic acid (p < 0.01) and flurbiprofen (p < 0.001) [18]. 

Due to limited resources, our study was only conducted in a single center, which significantly limited the generalizability of the study findings. Furthermore, the study could not explore subjective patient satisfaction due to language barrier and time restraints. Future research should focus on evaluating cost-effective therapies, comparing improvement in the quality of life with respect to different management options, and exploring a standard measure for evaluating patient satisfaction.

## Conclusions

The current study revealed that both oral tranexamic acid and combined oral contraceptive (COC) pills were equally effective in reducing the mean blood loss among patients with heavy menstrual bleeding (HMB). It was further found that the efficacy of both therapies was significantly higher in younger age groups. The efficacy of therapy was significantly reduced with the increasing age of the patient. Moreover, it was found that patients with leiomyomas benefitted more significantly from COC pills. There were no severe adverse effects reported in the study. However, future researches can explore the long-term side effects of both therapies.

In short, both therapies were comparable in terms of efficacy and safety. Heavy menstrual bleeding can negatively impact a woman, emotionally and physically. Therefore, it is encouraged that physicians use their expert judgment while prescribing either oral tranexamic acid or COC pills to patients with HMB.
